# Rhizosphere *Bacillus proteolyticus* Strain Enhances the *Eleutherococcus senticosus* (Rupr. & Maxim.) Maxim. Growth in Roots and Soil Nutrient Status While Enriching the Plant-Beneficial Bacteria in Rhizosphere

**DOI:** 10.3390/biology14121633

**Published:** 2025-11-21

**Authors:** Ye Zhang, Xiaoqing Tang, Jiaying Qi, Weixue Zhong, Xiaohui Li, Zhonghua Tang, Ying Liu, Dewen Li

**Affiliations:** 1 College of Chemistry, Chemical Engineering and Resource Utilization, Northeast Forestry University, Harbin 150040, China; zyzy1111@nefu.edu.cn (Y.Z.); 2023120862@nefu.edu.cn (X.T.); qjy@nefu.edu.cn (J.Q.); 2023120977@nefu.edu.cn (W.Z.); lxh@nefu.edu.cn (X.L.); tangzh@nefu.edu.cn (Z.T.); 2 Key Laboratory of Forest Plant Ecology, Ministry of Education, Northeast Forestry University, Harbin 150040, China; 3 Heilongjiang Provincial Key Laboratory of Ecological Utilization of Forestry-Based Active Substances, Harbin 150040, China

**Keywords:** bacterial community, *Eleutherococcus senticosus* (Rupr. and Maxim.), PGPB, root development, soil nutrient

## Abstract

This study aimed to investigate the promoting mechanisms of *Bacillus proteolyticus (B. proteolyticus)* on *Eleutherococcus senticosus* (Rupr. and Maxim.) Maxim. (*therm E. senticosus*) root development. Using *therm E. senticosus* seeds as experimental material, soil was drenched with *B. proteolyticus* suspensions at different concentrations *v/v* (water, 25%; 50%; 75%, and 100%). The results showed that the germination rate, root tip number (52 ± 2.97), total root length (23.7 ± 0.46 cm), and total root volume (57.36 ± 1.64 mm^3^) exhibited an initial increase, then a decrease after *B. proteolyticus* treatment (*p* < 0.05). Principal component analysis (PCA) indicated that the soil of *B. proteolyticus* at a 50% concentration was conducive to seed germination. Compared with CK, GC-MS analysis revealed that 16 differential primary metabolites were screened, primarily enriched in galactose metabolism, starch and sucrose metabolism, and TCA cycle pathways after 50% *B. proteolyticus* treatment. LC-MS analysis revealed that the contents of six main medicinal components were higher than those of CK, with the content of eleutheroside E being 2.62 times greater. In rhizosphere soil, the contents of NO_3_^−^-N and NH_4_^+^-N were promoted, and the abundance of *Gemmatimonadetes* was increased in bacterial communities. Correlation analysis revealed significant correlations between the abundance of *Gemmatimonadetes* and the contents of NO_3_^−^-N and NH_4_^+^-N, as well as between total root length and D-galactose content, suggesting that these relationships may contribute to the root growth. Therefore, the soil of *B. proteolyticus* at a 50% concentration could enhance both the biomass and medicinal value of cultivated *therm E. senticosus*. This study provided novel insight that *B. proteolyticus* would be expected to be developed as an effective microbial preparation, offering a sustainable strategy for its agricultural production.

## Simple Summary

This study explored how *Bacillus proteolyticus* (*B. proteolyticus*) helps the root development of *Eleutherococcus senticosus* (Rupr. and Maxim.) Maxim. (*therm E. senticosus*). The researchers used *therm E. senticosus* seeds and watered the soil with different concentrations of *B. proteolyticus* suspension. Results showed that after using *B. proteolyticus*, the seed germination rate, number of root tips, total root length and total root volume of *therm E. senticosus* first increased and then decreased. The 50% concentration of *B. proteolyticus* was best for seed germination. Compared with the control group, this concentration led to 16 different substances related to plant metabolism and an increase in the content of six main medicinal components. It also increased two types of nitrogen in the soil around roots and the amount of a specific kind of bacteria, which were all related to better root growth. This study found that 50% *B. proteolyticus* can promote *therm E. senticosus* growth, root development, and its medicinal quality. These bacteria can be made into a useful biological product. The study suggests using 50% *B. proteolyticus* suspension to grow *therm E. senticosus*, which can increase its yield and medicinal value, providing a sustainable way for its agricultural production.

## 1. Introduction

*Eleutherococcus senticosus* (Rupr. and Maxim.) Maxim. (*E. senticosus*) was a perennial deciduous shrub belonging to the *Araliaceae* family and the genus *Eleutherococcus* [1]. Its roots and rhizomes were used medicinally and have been widely utilized as a folk herbal medicine in northeastern China [2]. *Therm E. senticosus* was one of the Traditional Chinese Medicines (TCM) stipulated in the Pharmacopoeia of the People’s Republic of China, which plays a role in treating and preventing rheumatism, coronary heart disease, hyperlipidemia, and depression [3]. The unrestrained harvesting of wild *therm E. senticosus* has led to severe destruction of its wild resources and a sharp decline in its population, due to its extensive medicinal and economic value [4]. However, seeds of *therm E. senticosus* possess innate dormancy characteristics. Under natural conditions, the seeds require two years to achieve a mere 10% germination rate, which makes artificial cultivation challenging in compensating for the shortfalls of wild resources [5]. In recent years, a lot of studies have focused on enhancing the germination rate of ES seeds.

Plant growth-promoting bacteria (PGPB) were emerging as novel practices and successful biological solutions to promote seed germination and boost medicinal and agricultural plant growth, development, and production [6,7]. PGPB inoculation can reshape the plant-associated microbiome, enhancing connectivity between rhizosphere and endophytic microbial communities and potentially improving crop resilience [8]. While the potential of PGPB is well-recognized, studies specifically exploring their application in *therm E. senticosus* cultivation remain limited. *Bacillus thuringiensis* CAPE95 and *Paenibacillus polymyxa* CAPE238 have been shown to increase germination rates and germination indices in nasturtium (*Tropaeolum majus*) and horsetail casuarina (*Casuarina equisetifolia*) [9,10]. Furthermore, PGPB can significantly enhance the accumulation of minor metabolites in medicinal plants by modulating their secondary metabolism. The study suggested that the application of *Plesiomonas* bacteria provides interesting benefits to an increase in crocin and safranal content in *Crocus sativus* [11]. The *Bacillus sphaericus* CIMAP-A7, found in *Andrographis paniculata* (Burm. f.) Nees was involved in the biosynthesis and accumulation of secondary metabolites [12]. Previous research has demonstrated that PGPB can not only promote growth but also significantly enhance the accumulation of bioactive compounds. For instance, the co-application of mycorrhizal fungi and specific PGPB with vermicompost has been shown to substantially increase the total phenolic content and fruit yield in *Physalis alkekengi* L., highlighting the potential of microbial communities in enhancing both the quality and quantity of medicinal plant production [13]. Therefore, investigating the growth-promoting mechanisms of PGPB from medicinal plants was crucial for formulating effective cultivation strategies and enhancing the quality of its seedlings.

PGPB mediates the modulation of soil nutrient availability. Research reports indicate that PGPB can convert organic phosphorus compounds into phosphate in soil or transform insoluble inorganic phosphorus into soluble phosphorus to promote plant growth [14]. It was well known that PGPB could improve plant growth and change root conformation through direct mechanisms, including the synthesis of phytohormones and growth regulators such as auxin (indole-3-acetic acid (IAA)) [15]. This inoculation with phosphate-solubilizing bacteria ASR16 and ALR332 showed a significant growth improvement in *Medicago sativa* L. compared to the control, especially in plant height, biomass, and root length [16]. In addition, PGPB can promote soil nitrogen fixation, providing important nutrient elements for plants [17]. The current research was motivated by the isolation of a bacterial strain from the rhizosphere soil of two-year-old *E. senticosus*, which was later identified as *Bacillus proteolyticus*. Preliminary functional characterization revealed its promising plant growth-promoting abilities, leading to a systematic evaluation of its effects on the growth and metabolism of its host plant. We studied the effect of *B. proteolyticus* from *therm E. senticosus* rhizosphere on its seed germination and analyzed the related physiological mechanisms combined with the rhizosphere soil nutrient and bacterial community structure. Therefore, investigating the growth-promoting mechanisms of PGPB from medicinal plants was crucial for formulating effective cultivation strategies and enhancing the quality of its seedlings.

## 2. Materials and Methods

### 2.1. Bacterial Strain

Strain identification was performed based on morphological, physiological, and biochemical characteristics following Bergey’s Manual of Determinative Bacteriology [18]. The physiological and biochemical characteristics following standard microbiological methods were employed as follows: Gram staining, catalase test, oxidase test, gelatin hydrolysis test, starch hydrolysis test, methyl red test, Voges–Proskauer (V–P) test, nitrate reduction test, indole production test, and hydrogen sulfide (H_2_S) production test. The functional traits were assessed using the following assays: the nitrogen-free Jensen’s medium method for nitrogen-fixation, the Pikovskaya’s broth method for phosphate solubilization, and the Salkowski’s reagent method for indole-3-acetic acid (IAA) production quantification.

Genomic DNA extraction was conducted using a proteinase K lysis protocol. PCR amplification was carried out with universal bacterial 16S rDNA primers, forward primer 5′-AGAGTTTGATCCTGGCTCAG-3′ and reverse primer 5′-AAGGAGGTGATCCAGCC-3′. The PCR amplification system and reaction conditions are detailed in Table 1. Amplified 16S rDNA products were purified and subsequently sent to Shanghai Majorbio Bio-pharm Technology Co., Ltd. (Shanghai, China) for sequencing. The obtained sequences were submitted to the NCBI database for Blastn analysis (Table 1).

### 2.2. B. proteolyticus Suspension and Soil Preparation

A single colony of *B. proteolyticus* was inoculated into 200 mL of liquid LB medium and cultured at 37 °C with shaking at 200 rpm for 8–10 h until the OD_600_ reached 0.8, after which the bacterial cells were collected by centrifugation at 12,000 r/min for 10 min. The pellets were then resuspended in sterile distilled water and diluted to concentrations of 25%, 50%, 75%, and 100% (*v*/*v*), designated as T25, T50, T75, and T100. Using sterile distilled water as CK. The resulting suspensions were stored at 4 °C.

The potted soil used in this study was peat soil. The scope was conducted in a pot with an upper diameter of 54.5 mm, a bottom diameter of 28.5 mm, and a depth of 5 cm. Each treatment consisted of 5 pots, with 300 seeds shown per pot. Each pot was irrigated with 825 mL of bacterial suspension after seeding.

### 2.3. Plant Materials

*Therm E. senticosus* seeds were purchased from the Northern Medicinal Plant Planting Base, Yichun City, Heilongjiang Province, and cultivated in the greenhouse of the Key Laboratory of Forest Plant Ecology, Ministry of Education at Northeast Forestry University (Harbin City, Heilongjiang Province, China). The different concentrations of suspensions are drenched in soil.

### 2.4. Determination of Seed Germination and Root Growth

Germination rates ((Germinated seeds/Total tested seeds) × 100%) were recorded at five-day intervals post-sowing until day 50. The germination rate was calculated by dividing the number of germinated seeds by the total number of seeds shown (300). At 50 days post-treatment, leaf area was measured using a portable leaf area meter (CI-203; CID Bio-Science, Inc., Camas, WA, USA). Plant height and stem length were determined using a ruler. Fresh weight and dry weight were measured with an analytical balance (FA224C; LICHEN, Shanghai, China). For root character analysis, five uniformly growing seedlings per treatment were randomly selected. Roots were thoroughly rinsed and scanned using a scanner (IN-GX02; LAIYIN, WeiFang City, China). Root length, surface area, volume, and average diameter were quantified from digital images.

### 2.5. Determination of Metabolic Characteristics in Roots

The determination of primary metabolites followed the methodology described by Liu [19] using an Agilent 7890A-5975C GC-MS system (7890A-5975C; Agilent, Newark, DE, USA). Fresh roots from the CK and treatment (T50) were selected for metabolic analysis. For primary metabolic profiling, 0.09 g of each sample was used. Plant tissues were snap-frozen in liquid nitrogen and homogenized into a fine powder. Metabolites are then extracted using a mixed solvent system (methanol-water-chloroform), followed by centrifugation. The supernatant was collected, concentrated, and reconstituted in a suitable solvent for subsequent GC-MS analysis. This analysis was conducted using a DB-5MS capillary column in split mode, with a 1 μL sample injection and helium as the carrier gas. After a 6.35 min solvent delay, mass spectra were acquired in full-scan mode (*m*/*z* 50–500) at a scan rate of 12.5 spectra/s.

Major medicinal active constituents and phenolic compounds in ES were analyzed according to Fu’s protocol [20] using an ACQUITY UPLCXevoG2-SQToFMS system (6890GCMS; Waters Technologies, Shanghai, China). Fresh roots (0.5 g) were frozen in liquid nitrogen. The material was ground into a fine powder, followed by extraction with a methanol-water solvent system. After centrifugation, the supernatant was collected, concentrated under a nitrogen stream, and reconstituted in appropriate solvents for subsequent LC-MS analysis. Separation was achieved on a C18 column (2.1 mm × 100 mm, 1.6 μm) with mobile phase, 62% water (solvent A) and 38% methanol (solvent B) at 0.25 mL·min^−1^. Column temperature was maintained at 25 °C with a 5 μL injection volume. Reference standards were purchased from the Aladdin official website.

### 2.6. Determination of Soil Characteristics

According to Ru’s method [21], Soil pH was determined by using a pH meter (PhSJ-5T; Leici, Shanghai, China), and soil electrical conductivity (EC) was determined by using a conductivity meter (DDS-307; Leimagnet, Shanghai, China). Soil ammonium nitrogen (NH_4_^+^-N) was determined by Nanocolorimetry, and nitrate nitrogen (NO_3_^−^-N) by phenol-disulphonic acid, and available phosphorus (AP) by the Olsen Method.

### 2.7. Determination of Rhizosphere Soil Bacterial Communities

Rhizosphere soil DNA from the CK and T50 treatments was extracted and analyzed. Nucleic acid extraction and sequencing were outsourced to Shanghai Personalbio Biotechnology Co., Ltd. (Shanghai, China) Genomic DNA was extracted using the Mag Beads Fast DNA Kit for Soil (116564384; MP Biomedicals, Santa Ana, CA, USA). Sequencing was performed on an Illumina HiSeq 2500 platform (Illumina Inc., San Diego, CA, USA). Raw sequencing reads were processed through paired–end merging, quality filtering, and chimera removal to obtain high-quality sequences (Clean Tags). Subsequently, Clean Tags were clustered into Operational Taxonomic Units (OTUs) at 97% similarity threshold using Usearch9. Analysis of species abundance distribution was visualized through Venn diagrams and QIIME 1 pipelines. All bioinformatic analyses were conducted on the Personalbio Cloud Platform (https://www.genescloud.cn/ accessed on 6 February 2025).

### 2.8. Statistical Analysis

Data processing was performed using SPSS 26.0 and Microsoft Excel 2021. Differences between treatment groups were assessed by one-way analysis of variance (ANOVA) at *p* < 0.05 significance level. Comprehensive scores across treatments were calculated through principal component analysis (PCA). Figures were generated with Origin 2021, with data presented as mean ± standard deviation (mean ± SD).

## 3. Results

### 3.1. Identification of Rhizosphere B. proteolyticus

**Figure 1:**
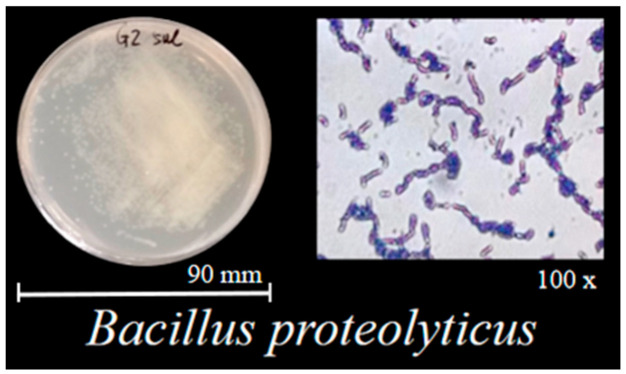
Morphological characteristics and Gram staining results of *B. proteolyticus* strain.

The *B. proteolyticus* strain used in this study was previously isolated from the rhizosphere soil of two-year-old *therm E. senticosus*. Combined with colonial morphology, the colonies exhibited a cream-colored, opaque appearance, predominantly circular with regular margins. Surface characteristics included glossiness without wrinkles, moist texture consistent with typical bacterial colony morphology (Figure 1); the strain was preliminarily identified as *B. proteolyticus*. PCR amplification and sequencing revealed 100% similarity to *B. proteolyticus* strain MCCC 1A00365.

Physiological and biochemical assays identified the strain as Gram–positive (Figure 1), positive for catalase, oxidase, gelatin hydrolysis, starch hydrolysis, methyl red test, nitrate reduction, and indole production, but negative for Voges–Proskauer (V–*p*) reaction and H_2_S production. Functional characterization might demonstrate the action of its nitrogen-fixing capacity, phosphate solubilization (22.79 ± 0.92 μg/mg), and IAA production (14.99 ± 0.83 mg/L) (Table 2). Based on these findings, the strain was identified as a PGPB with multifunctional activities.

### 3.2. Promotion of E. senticosus Seed Germination and Root Growth by B. proteolyticus

**Figure 2:**
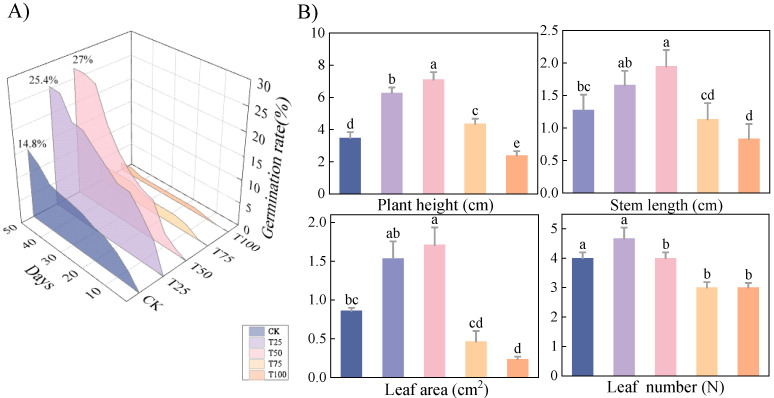
*Therm E. senticosus* growth characteristics after different *B. proteolyticus* treatments. (**A**) Seed germination rate, (**B**) growth parameters of shoots. Note: different lowercase letters indicate significant differences (*p* < 0.05) among treatments. Differences between treatment groups were assessed by one-way analysis of variance (ANOVA) at *p* < 0.05 significance level.

As the *B. proteolyticus* concentration increased, the germination rate of *therm E. senticosus* seeds initially rose, then declined. At day 50 post-treatments, the highest germination rate (27%) was achieved after the T50 treatment (Figure 2A), and the seedlings’ height gradient followed: T50 > T25 > T75 > CK > T100; the maximal height (7.10 ± 0.47 cm) was exhibited significantly (*p* < 0.05). Both parameters were significantly enhanced compared to all treatments except T25 (*p* < 0.05), with stem length 1.5-fold greater than CK after T50 treatment (Figure 2B). While germination rate, plant height, stem length, and leaf area were optimally promoted, it showed limited efficacy in increasing leaf number after T50 treatment.

**Figure 3:**
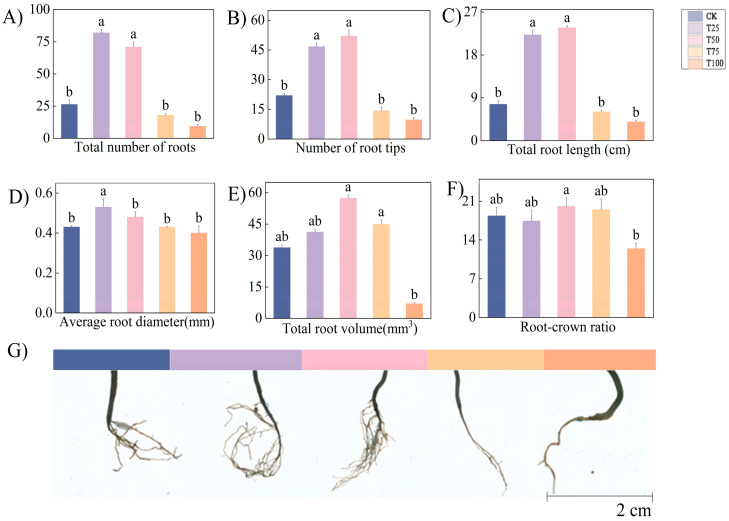
Root growth characteristics of *therm E. senticosus* after different *B. proteolyticus* treatments. (**A**) Total number of roots, (**B**) number of root tips, (**C**) total root length, (**D**) average root diameter, (**E**) total root volume, (**F**) root-crown ratio, (**G**) root system phenotype images. Note: different lowercase letters indicate significant differences (*p* < 0.05) among treatments. Differences between treatment groups were assessed by one-way analysis of variance (ANOVA) at *p* < 0.05 significance level.

After T50 treatment, *therm E. senticosus* exhibited the highest values among all treatments for root tip number (52 ± 2.97), total root length (23.7 ± 0.46 cm), total root volume (57.36 ± 1.64 mm^3^), and root-to-shoot ratio (20.10 ± 1.54). Compared to CK, T75, and T100 treatments, there was a demonstrated significant enhancement effect (*p* < 0.05) after T50 treatment. The average root diameter after T25 (0.53 ± 0.04 mm) was significantly greater than other treatments (*p* < 0.05), while differences among other treatments were non-significant (*p* < 0.05). Lower concentrations of *B. proteolyticus* more effectively promoted root system development in ES but exerted minimal effects on root-to-shoot ratio and root diameter (Figure 3).

**Figure 4:**
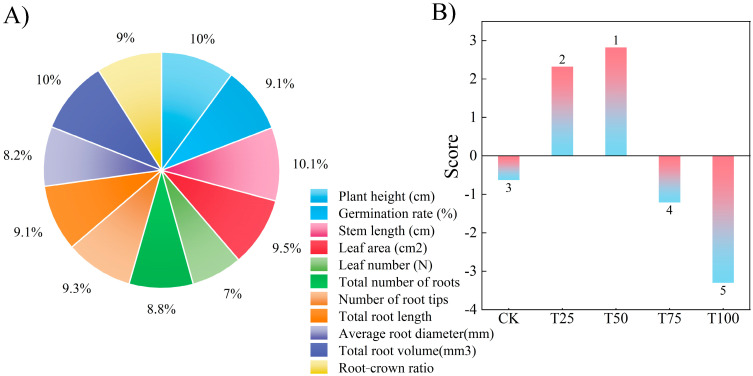
Principal component analysis. (**A**) the weights of the 11 measured indices, (**B**) the comprehensive scores.

Principal component analysis (PCA) was performed on 11 indices across five treatments in this study, and the results are shown in Figure 4 and Table 3. The weights of the 11 measured indices were 10.03%, 9.11%, 10.06%, 9.49%, 6.99%, 8.75%, 9.29%, 9.09%, 8.17%, 10.04%, and 8.97% (Figure 4A). The comprehensive scores of each treatment, calculated by principal component analysis, are presented in Figure 4A, with the ranking of comprehensive scores as follows: T50 > T25 > CK > T75 > T100. The results indicated that the germination and growth status of ES after the T50 treatment were the best among all treatments (Figure 4B).

### 3.3. Analysis of Metabolic Characteristics of Root

**Figure 5:**
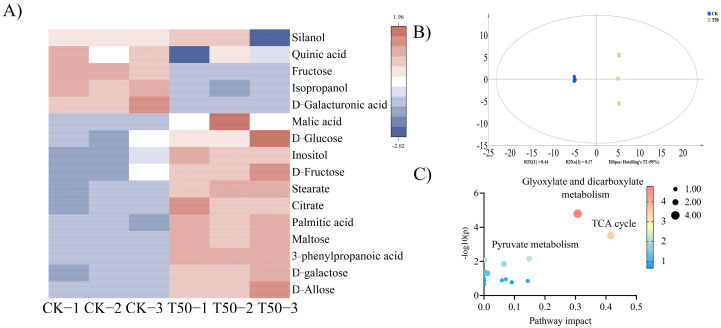
The primary metabolites analysis after different concentrations of *B. proteolyticus* treatments in *therm E. senticosus* roots. (**A**) cluster analysis plot, (**B**) OPLS-DA scores plot, and (**C**) pathway enrichment analysis of differential metabolites.

A total of 60 specific primary metabolites were identified. Based on the results of OPLS-DA analysis, metabolites with a VIP value greater than one and a significant difference *p*-value between groups less than zero point zero five were screened out. A total of 16 differential metabolites between the two treatments were screened out, which were classified into six acids, six sugars, three alcohols, and one phenylpropanoid. The six acids were D-galacturonic acid, quinic acid, citric acid, malic acid, palmitic acid, and stearic acid; the six sugars were d-galactose, maltose, D-psicose, D-fructose, D-glucose, and sucrose; the three alcohols were silanol, inositol, and isopropanol; the one phenylpropanoid was phenylpropionic acid. Compared with CK, the contents of citric acid, silanol, phenylpropionic acid, malic acid, inositol, D-galacturonic acid, sucrose, quinic acid, D-glucose, palmitic acid, stearic acid, D-fructose, D-galactose, and isopropanol in T50 are significantly increased (Figure 5A). After treatment with *B. proteolyticus* solution, the differences in primary metabolic pathways of ES root were concentrated in galactose metabolism, starch and sucrose metabolism, TCA cycle, glyoxylate and dicarboxylate metabolism, and pyruvate metabolism. Among them, the differential metabolites (citric acid, silanol, phenylpropionic acid, malic acid, and inositol) after T50 treatment were mainly concentrated in the TCA cycle, glyoxylate and dicarboxylate metabolism, and pyruvate metabolism (Figure 5C). The accumulation of carbohydrates in *therm E. senticosus* was promoted significantly after *B. proteolyticus* treatment.

**Figure 6:**
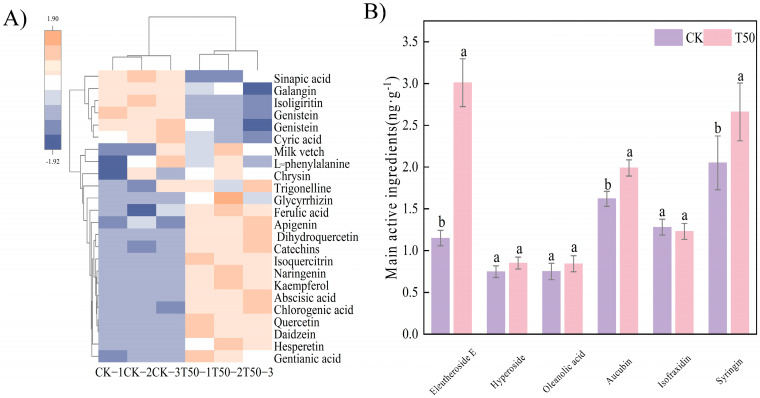
Analysis of phenolic compounds and main medicinal components after T50 treatment. (**A**) visualization heatmap of phenolic compounds, (**B**) contents of main medicinal components. Note: different lowercase letters indicate significant differences (*p* < 0.05) among treatments. Differences between treatment groups were assessed by one-way analysis of variance (ANOVA) at *p* < 0.05 significance level.

Metabolomic analysis of the roots revealed distinct accumulation patterns of both primary and secondary metabolites compared to the control. (Figure 6A). Compared with CK, there were higher contents of galangin, quercetin, abscisic acid, apigenin, chlorogenic acid, dihydroquercetin, kaempferol, isoquercitrin, glycyrrhizin, trigonelline, hesperetin, genistein, and catechins (Figure 6A). The contents of eleutheroside E, aucubin, and syringin in roots were significantly higher than those in CK (*p* < 0.05) (Figure 6B). After treatment, the content of secondary metabolites in ES was significantly (*p* < 0.05) accumulated.

### 3.4. Effects on Soil Biochemical Characteristics

**Figure 7:**
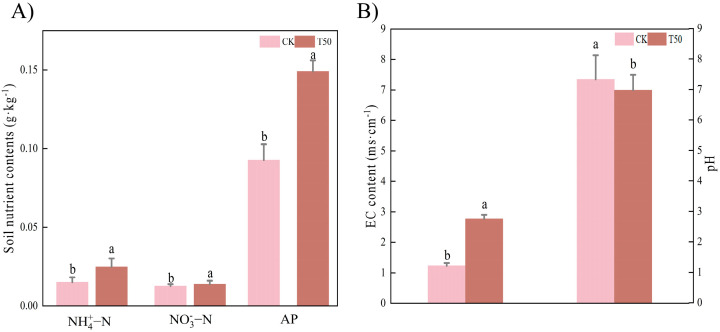
Soil characteristics analysis after T50 treatment of the rhizosphere soil of *therm E. senticosus* seedlings. (**A**) soil nutrient contents, (**B**) pH, and EC. Note: different lowercase letters indicate significant differences (*p* < 0.05) among treatments. Differences between treatment groups were assessed by one-way analysis of variance (ANOVA) at *p* < 0.05 significance level.

As shown in Figure 7A, there were higher contents of soil ammonium nitrogen (NH_4_^+^-N), nitrate nitrogen (NO_3_^−^-N), and available phosphorus (AP) compared to CK after T50 treatment. The pH of CK was significantly (*p* < 0.05) higher than that of treatment, while the EC was significantly higher than that of CK (Figure 6B). Treatment with the bacterial suspension significantly (*p* < 0.05) increased the nutrient content in the soil.

### 3.5. Correlation Analysis

**Figure 8:**
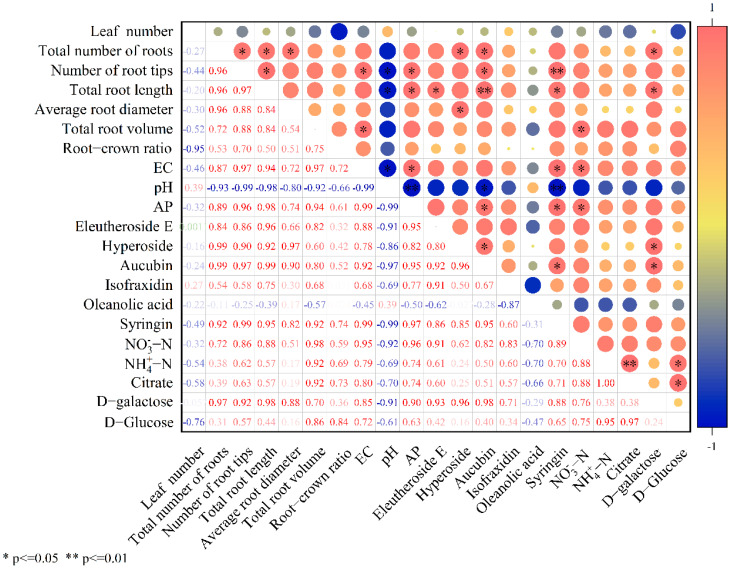
Correlation coefficients between growth characteristics, soil nutrient contents, and main medicinal components of *therm E. senticosus* after different concentrations of *B. proteolyticus* treatments.

The contents of citrate and D-Glucose were significantly (*p* < 0.05) positively correlated with NH_4_^+^-N content. D-galactose was significantly (*p* < 0.05) positively correlated with total root length. There was a significant (*p* < 0.05) positive correlation between NO_3_^−^-N content and total root volume. AP content was significantly (*p* < 0.05) positively correlated with total root length and number of root tips. The content of eleutheroside E, aucubin, and syringin was significantly positively correlated with total root length and number of root tips (*p* < 0.01) (Figure 8). The contents of AP influenced secondary metabolite accumulation in *therm E. senticosus*.

### 3.6. Analysis of the Rhizosphere Soil Bacterial Community Diversity and Community Composition

After filtering the raw sequences, we obtained statistically significant species counts for each taxonomic rank in each sample. There was a higher total number of bacterial groups OTUs in the rhizosphere soil of therm *E. senticosus* after the T50 treatment than after CK treatment. Statistical tables of bacterial species can be acquired by consulting the Silva and UNITE databases (Table 3). The bacterial diversity after T50 treatment was higher than that after CK at all levels except the domain level.

**Figure 9:**
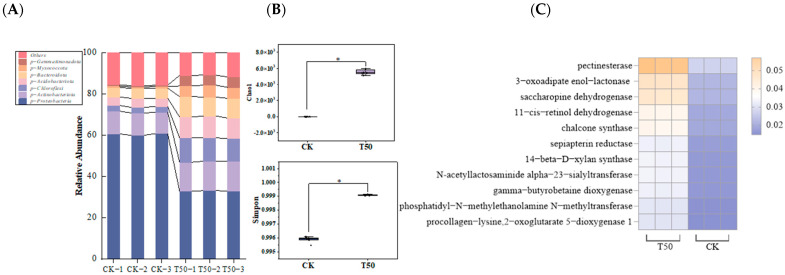
Analysis of soil bacterial community characteristics in *therm E. senticosus* after T50 treatment. (**A**) Primary metabolism of bacterial communities, Simpson and Chao1index, (**B**) bacterial community composition, and (**C**) functional divergence. * *p* ≤ 0.05.

*Proteobacteria* were the dominant phylum after both CK and T50 treatments, accounting for 60.51 ± 0.01% and 33.07 ± 0.01% of the communities. There were significantly higher relative abundances of *Gemmatimonadetes*, *Myxococcota*, *Bacteroidetes*, *Acidobacteria,* and *Chloroflexi* after T50 treatment (Figure 9A). The abundance of *Gemmatimonadetes* was 8.33 times higher than that of CK. As a comprehensive measure of the α-diversity index, the Simpson index and Chao index showed a significant difference between CK and T50 treatments (Figure 9B). PICRUST2 was used to predict the potential functions of the bacterial community in rhizosphere soil. The results showed that the primary metabolic functions of the rhizosphere soil bacterial communities in CK and T50 mainly included the following nine categories: interconversion of pentose and glucuronate, degradation of aromatic compounds, lysine biosynthesis, retinol metabolism, flavonoid biosynthesis, folate biosynthesis, amino sugar and nucleotide sugar metabolism, lysine degradation, and glycerophospholipid metabolism, all of which were higher than those in CK (Figure 9C). After T50 treatment, microbial community richness was enhanced in the rhizosphere soil, and primary metabolic functions among microorganisms were altered.

**Figure 10:**
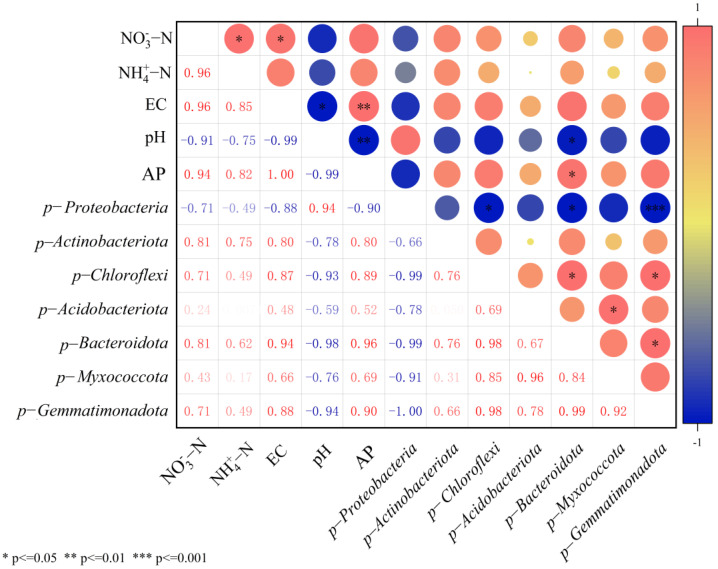
Correlation coefficients between soil nutrient contents and bacterial community composition of rhizosphere soil in *therm E. senticosus* seedlings after T50 treatment.

Correlation analysis demonstrated that all bacterial phyla showed differential positive correlations with NO_3_^−^-N, NH_4_^+^-N, AP, and EC except *Proteobacteria* after T50 treatment, while exhibiting negative correlations with pH. Consequently, the T50 treatment resulted in significantly higher nutrient content in the soil of *therm E. senticosus* (Figure 10).

## 4. Discussion

In recent years, there has been a surge of interest in investigating the promotional effects of plant growth-promoting bacteria (PGPB) on seed germination, growth, and metabolic processes of medicinal plants [22]. PGPB accelerates the growth of medicinal plants and enhances their nutrient absorption efficiency, as they could intervene in and coordinate their growth and nutritional metabolic pathways [23]. Studies have shown that an appropriate bacterial suspension concentration is more conducive to plant growth [24]. Therefore, we established gradient concentrations of the bacterial suspension of *B. proteolyticus* and conducted a Principal Component Analysis (PCA) based on the growth indicators of *therm E. senticosus*, which demonstrated that the T50 treatment yielded the most pronounced promoting effect on the plant growth. Studies have shown that PGPB treatment can increase the germination rate of tomato and *Seriphidium transiliense* seeds, as well as enhance the aboveground biomass and rhizome elongation rate of *Zostera marina* L. [25]. In our study, inoculation with *B. proteolyticus* significantly promoted the germination rate and growth indicators of *therm E. senticosus*, resulting in plant height, stem length, and leaf area that were 2.05, 1.53, and 1.98 times that of CK, respectively, and a 12.2% increase in germination rate (Figure 1). This biosynthesis of IAA can promote plant growth and development and enhance plant metabolic activity [26,27]. We analyzed the PGP properties of *B. proteolyticus* using in vitro assays to demonstrate its ability to produce IAA, solubilize phosphate (Table 1), indicating that the impact of PGPB on plant development was associated with IAA production [28]. PGPB play a pivotal role in stimulating the production of plant growth regulators, fortifying plant defenses, and mitigating abiotic stress [29,30,31].

The use of microorganisms as green fertilizers to enhance the active ingredients and cultivation yields of medicinal plants is currently a hot research topic [32]. The secondary metabolites of plants were not only crucial to their own defense mechanism but also a source of pharmacologically active components [33,34]. In our study, drenching with *B. proteolyticus* significantly increased the content of secondary metabolites in *therm E. senticosus* seedlings, such as eleutheroside E and phenolic compounds. Similar results have been reported in other studies on medicinal plants. LIMA found that inoculation with *C. etunicatum* or *G. albida* increased the contents of total leaf phenols and tannins in *C. leptophloeos*. *Actinomadura roseola* could promote the upregulation of rutin and hesperetin in *Tetrastigma hemsleyanum* [35]. Three main active components, including volatile oils in Asarum sieboldii, showed a good correlation with the rhizosphere bacterium *Rhodanobacter* [36]. The biologics released by microorganisms have also been shown to effectively stimulate the production of key host plant enzymes responsible for secondary metabolite synthesis [37,38,39]. The ability of PGPB to reshape the root-associated microbiome and enhance crosstalk between rhizospheric and endophytic microbial communities may also contribute to the improved metabolic profiles observed in our study, underscoring a potential microbiome-mediated mechanism [4].

Patani argues that the *Bacillus genus* can directly stimulate plant growth through enhancing nutrient availability, such as nitrogen fixation and phosphate solubilization [40]. In our study, the incorporation of *B. proteolyticus* significantly enhanced the physicochemical properties of the soil, particularly augmenting the availability of essential nutrients such as NO_3_^−^-N, NH_4_^+^-N, and AP (Figure 6). After T50 treatment, *Gemmatimonadetes* abundance was higher than that of CK. Analysis of soil bacterial α-diversity in this study showed that the richness of the bacterial community in the rhizosphere soil of *therm E. senticosus* decreased after irrigation with *B. proteolyticus*, which was similar to the findings of Li [41]. They found that PGPB + SMS treatment significantly increased the abundances of *Bacillus* and *Pseudomonas*, which might be attributed to the limited ability of single PGPB irrigation to alter soil microbial community structure, whereas mixed bacterial inoculants enhanced the stability of the soil microbial community in *Phyllostachys edulis* [42]. Studies have shown that *Gemmatimonadetes* abundance exhibits a positive correlation with total carbon, nitrogen, and phosphorus in soil. The abundance of *Gemmatimonadetes* demonstrates strong metabolic and degradation capabilities, playing a crucial role in nutrient cycling and potentially enhancing plant nutrient utilization efficiency [43,44]. Inoculation with PGPB can well fix gaseous nitrogen in the air [16] and simultaneously increase the number of nitrogen-fixing bacteria in the soil, as well as decompose nitrates and nitrites in the soil, thereby increasing soil nitrogen levels [45]. Meanwhile, the contents of NO_3_^−^-N, NH_4_^+^-N, and AP after irrigation with *B. proteolyticus* were also significantly increased, which was consistent with the findings of Zhang [46]. They suggested that inoculation with PGPB can effectively convert unavailable forms of soil nutrients into available ones and promote the efficient absorption and utilization of nutrients by plants. The content of AP increased significantly after treatment, which was consistent with the conclusion of Liu [47]. The possible reason was that PGPB secretes inorganic and organic acids to dissolve insoluble phosphorus or upregulate the expression of phosphate transporters to enhance plant absorption and utilization of phosphorus [47]. Such a biofertilizer could serve as an environmentally friendly alternative to partial chemical fertilization, supporting sustainable agricultural practices.

## 5. Conclusions

The *B. proteolyticus* strain in this study functioned as a multifunctional PGPB, exhibiting capabilities for nitrogen fixation, phosphorus solubilization, and IAA biosynthesis. This study indicated that *B. proteolyticus* had a significant promoting effect on seed germination, root growth, and metabolism of *therm E. senticosus*. It increases the contents of AP, NO_3_^−^-N, and NH_4_^+^-N in soil through nitrogen fixation and phosphorus solubilization, and secretes IAA to promote the development and growth of *therm E. senticosus* roots for better nutrient absorption. Differences were observed in primary metabolic pathways, including galactose metabolism, starch and sucrose metabolism, TCA cycle, glyoxylate and dicarboxylate metabolism, and pyruvate metabolism. Additionally, the contents of the main medicinal active components accumulated through secondary metabolism were significantly increased. From a practical perspective, we recommend developing this strain into a specialized microbial inoculant for *therm E. senticosus* cultivation. Future research should focus on the following: validating the strain’s efficacy under field conditions, elucidating the molecular mechanisms underlying their role in host metabolic reprogramming.

## Figures and Tables

**Table 1:** PCR amplification system and reaction conditions.

Reagent Composition	Volume	Thermal Cycling Condition	Temperature	Time
5xBuffer	10 μL	Initial Denaturation	94 °C	2 min
dNTP (10 mM)	1 μL	Denaturation	94 °C	30 s
Phusion High-Fidelity DNA Polymerase	1 μL	Annealing	56 °C	30 s
F/R primer (10 μM)	1 μL	Extension	72 °C	90 s
Template DNA	1–3 μL	27 Cycles	Hold	
ddH_2_O	Supplement to 50 μL	Final Extension	72 °C	5 min

**Table 2:** Plant growth-promoting attributes of inoculum strains.

*Bacillus proteolyticus*	Fostering Capacity
Phosphate solubilization	22.79 ± 0.92 μg/mg
IAA production	14.99 ± 0.83 mg/L
Atmospheric nitrogen fixation	+

**Table 3:** The number of bacterial communities in rhizosphere soil.

	Bacteria OTU Number	Domain	Phylum	Class	Order	Family	Genus	Species
CK	10,240	1	26	65	144	232	368	149
T50	18,813	1	37	96	210	317	471	163

## Data Availability

Data will be made available on request.
